# Comprehensive appraisal of lung function in young COPD patients: a single center observational study

**DOI:** 10.1186/s12890-024-03165-9

**Published:** 2024-07-24

**Authors:** Lunfang Tan, Yun Li, Zhufeng Wang, Zihui Wang, Shuyi Liu, Junfeng Lin, Jinhai Huang, Lina Liang, Kang Peng, Yi Gao, Jinping Zheng

**Affiliations:** Guangzhou National Laboratory, National Center for Respiratory Medicine, National Clinical Research Center for Respiratory Disease, State Key Laboratory of Respiratory Disease, Institute of Respiratory Health, the First Affiliated Hospital of Guangzhou Medical University, No.151 Yanjiang Road, Guangzhou, Guangdong 510120 China

**Keywords:** Young COPD, Respiratory symptoms, Lung function, Diffusing capacity, Lung volume

## Abstract

**Purpose:**

The present study aimed to investigate the clinical characteristics and lung function impairment in young people diagnosed with chronic obstructive pulmonary disease (COPD).

**Patients and methods:**

We retrospectively enrolled patients with COPD who underwent symptom assessment and comprehensive pulmonary function tests at the First Affiliated Hospital of Guangzhou Medical University between August 2017 and March 2022. The patients were categorized into two groups based on age: a young COPD group (aged 20–50 years) and an old COPD group (aged > 50 years).

**Results:**

A total of 1282 patients with COPD were included in the study, with 76 young COPD patients and 1206 old COPD patients. Young COPD patients exhibited a higher likelihood of being asymptomatic, lower rates of smoking, and a lower smoking index compared to old COPD patients. Although young COPD patients had higher median post-bronchodilator forced expiratory volume in 1 s (post-BD FEV_1_) (1.4 vs.1.2 L, *P* = 0.019), diffusing capacity of the lung for carbon monoxide (D_L_CO) (7.2 vs. 4.6, *P*<0.001), and a lower median residual volume to total lung capacity ratio (RV/TLC) compared to their older counterparts, there were no differences observed in severity distribution by GOLD categories or the proportion of lung hyperinflation (RV/TLC%pred > 120%) between two groups. Surprisingly, the prevalence of reduced D_L_CO was found to be 71.1% in young COPD, although lower than in old COPD (85.2%).

**Conclusion:**

Young COPD showed fewer respiratory symptoms, yet displayed a similar severity distribution by GOLD categories. Furthermore, a majority of them demonstrated lung hyperinflation and reduced D_L_CO. These results underscore the importance of a comprehensive assessment of lung function in young COPD patients.

**Supplementary Information:**

The online version contains supplementary material available at 10.1186/s12890-024-03165-9.

## Introduction

Chronic obstructive pulmonary disease (COPD), characterized by persistent respiratory symptoms and airflow limitation, is usually considered a disease of the elderly and always diagnosed in the population over 60 years old [1]. However, growing evidence has shown that COPD also occurs in younger people. For example, a national cross-sectional study in China reported that the age-standardized prevalence of COPD was 1.4% in the 20–29 age group, 3% in the 30–39 age group, and 5.1% in the 40–49 age group [2]. And another Korean population-based cohort of 2236 randomly selected individuals aged 40–50 years, the prevalence of young patients with COPD was 4.2% [3]. The onset age of COPD is younger, and the prevalence of young COPD is gradually increasing. This trend suggests that we need to reassess the risk factors for COPD and pay more attention to the clinical characteristics of young individuals with COPD. Therefore, to facilitate more research on the early origins of COPD, the 2022 Global Initiative for Chronic Obstructive Lung Disease (GOLD) guidelines indicated that “COPD in young people” was defined as an age-dependent term for patients aged 20–50 years, who have a post-bronchodilator forced expiratory volume in 1 s (post-BD FEV_1_) to forced vital capacity (post-BD FVC) ratio (post-BD FEV_1_/FVC) < 0.7 regardless of the severity of airflow limitation [4]. 

Previous studies demonstrated that young COPD was dominated by GOLD 1 and GOLD 2 (96.7%), most of them might not go to the hospital due to the mild symptoms and airflow limitation, which resulted in insufficient attention paid to COPD in young people [5]. However, another study showed undiagnosed COPD in young people often had significant structural and functional abnormalities [6]. Moreover, young COPD patients have increased risks of exacerbations, comorbidity, and mortality compared with individuals without airflow limitation [3, 5–8]. Therefore, young individuals with COPD deserve widespread attention and research.

Pulmonary function testing (PFT) is a key tool for the assessment of COPD, which helps clinical doctors diagnose COPD and monitor disease progression by measuring spirometry, lung volume, and lung gas exchange capacity. In addition, PFT also helps to guide treatment plan adjustment, predict disease deterioration, and provide personalized management plans for patients. Therefore, it is necessary to comprehensively evaluate the lung function of young COPD patients. Although previous studies have investigated the lung function characteristics of young COPD, most of them only focused on spirometry. For example, Divo et al. demonstrated young COPD had a significantly higher FEV_1_ compared to old COPD, and 15%, 33%, 33%, and 0.3% in young COPD had GOLD 1, GOLD 2, GOLD 3, and GOLD 4, respectively, which was similar to old patients [7]. However, the other two studies showed that young COPD mainly presented GOLD1-2, and rare young subjects had more than severe airflow limitation [3, 5]. The proportion of subjects distributed among the GOLD categories remains unclear. Additionally, there are a lack of studies assessing the characteristics of lung volume and diffusing capacity in young COPD, and the changes of lung volume or diffusing capacity in such a specific population remain unknown.

Therefore, the objective of the present study was to comprehensively investigate lung function changes in young patients with COPD, including spirometry, bronchodilator responsiveness, lung volumes, and diffusing capacity. It is of interest to bring insight into the pulmonary function impairment of young COPD and provide new perspectives for future research, especially in exploring the disease mechanisms and early diagnosis of young COPD.

## Materials and methods

The study protocol was performed according to the Declaration of Helsinki and approved by the First Affiliated Hospital of Guangzhou Medical University Ethics Committee (ES-2023-140-01). Informed consent was obtained from all subjects. Information of all patients was kept confidential.

### Study population

Out-patients or inpatients with COPD were retrospectively enrolled from the National Center for Respiratory Medicine, National Clinical Research Center for Respiratory Disease, and the First Affiliated Hospital of Guangzhou Medical University from August 2017 to March 2022. According to the guideline of the GOLD 2022, our study defined young COPD as operationally by a post-BD FEV_1_/ FVC < 0.7 in patients aged 20–50 years, while those older than 50 years of age were defined as old COPD [4]. The enrolled patients should meet the criteria of completing comprehensive assessments of lung function tests and a routine questionnaire conducted before lung function tests (Supplementary Table 1). The questionnaire included age, sex, symptom assessment (cough, sputum, dyspnea), modified Medical Research Council (mMRC), self-reported smoking history, air pollution exposure, self-reported respiratory history and so on. Lung function tests included spirometry, bronchodilator responsiveness (BDR) testing, lung volume measurements, and diffusing capacity of the lung for carbon monoxide (D_L_CO). Excluded criteria were as follows: (1) those with ambiguous diagnosis; (2) those diagnosed with asthma or obliterative bronchiolitis (meeting three major criteria and at least one minor criteria. The major criteria include FEV_1_/FVC < 0.7, negative bronchodilator responsiveness, and excluding respiratory infections [perform tests such as high-resolution CT and microbial culture based on clinical symptoms]. The minor criteria include the presence of chronic graft-versus-host disease, expiratory HRCT showed air trapping, dilatation and thickening of small airways, and RV%pred > 120%); (3) those with missing important parameters such as age, weight, FEV_1_, FVC, FEV_1_/FVC and D_L_CO; (4) those with extreme values, i.e. values outside of 1.5 * interquartile range (IQR). For the subjects who have performed multiple BDR tests, only the initial report was selected.

### Pulmonary function tests

The PFT equipment (Jaeger Masterscreen Body, BD, Franklin Lakes, NJ, USA; Cosmed PFT Quark, COSMED, The Metabolic Company, Rome, Italy) met the criteria of the American Thoracic Society and the European Respiratory Society (ERS/ATS). Spirometry, lung volume measurements, and single-breath diffusing capacity were conducted by trained and skilled technicians in accordance with ERS/ATS [9–12] and Pulmonary Function Group, Respiratory Diseases Society of Chinese Medical Association, [13–15] with standardization of protocols and quality control procedures across the clinical site. Only subjects with tests judged acceptable and reproducible were included. Each subject inhaled 400 µg of salbutamol (Ventolin, Glaxo Wellcome Products, France) via metered dose inhaler and repeated spirometry after 20–30 min. The lung volumes were measured by body plethysmography. D_L_CO was measured by the single-breath method and was corrected for hemoglobin in those with blood routine examinations. Predicted values of spirometry, lung volume, and D_L_CO were calculated from the reference equations published by Zheng and Zhong, [16] Stocks et al [17] and ATS, [18] respectively. All the lung function tests were conducted within a month.

### Lung function indices and variable definitions

Spirometry, lung volume measurement, and D_L_CO were standardized as percentages of predicted values as described previously. The regular indices included FEV_1_, FEV_1_%pred, FVC, FVC%pred, FEV_1_/FVC, maximal-mid expiratory flow (MMEF), MMEF%pred, RV/TLC, RV/TLC%pred, D_L_CO, and D_L_CO%pred. According to the 2005 ERS/ATS criteria, the positive BDR met the change of ≥ 12% and 200 mL in FEV_1_ and/or FVC between the optimal value of baseline and post-bronchodilator. The grades of COPD conformed to GOLD guidelines, GOLD 1: FEV_1_%pred ≥ 80%, GOLD 2: 50% ≤ FEV_1_%pred < 80%, GOLD 3: 30% ≤ FEV_1_%pred < 50%, and GOLD 4: FEV_1_%pred < 30% [19]. An abnormal ratio of RV/TLC was considered pathological and pulmonary hyperinflation was defined as RV/TLC%pred > 120% [20]. The severity of reduced D_L_CO was assessed by D_L_CO%pred with 3 critical values of 80%, 60%, and 40%, namely normal: D_L_CO%pred ≥ 80% or LLN, mild: 60% ≤ D_L_CO%pred < 80%, moderate: 40% ≤ D_L_CO%pred < 60%, severe: D_L_CO%pred < 40% [21]. 

### Sample size imbalance

We supposed that the sample size of old COPD was much larger than young COPD, which might lead to bias in results. In this study, a total of 76 young COPD patients and 1206 old COPD patients were included. To ensure a balanced distribution of sample sizes between the two groups, a random under-sampling method was implemented using SPSS. Each old COPD patient was assigned a random number, and subsequently, 152 individuals were randomly selected from this group, resulting in a balanced ratio of 1:2 between the young and old COPD patients. We would compare the differences between analysis results before and after balancing the data, and the more reliable results would be mainly discussed.

### Statistical analysis

All analyses were performed using SPSS version 25. Continuous variables were presented as the mean and standard deviation (SD) for normally distributed data, or the median and interquartile range (IQR) for non-normally distributed data. Categorical variables were presented as percentages. Differences between young COPD and old COPD patients were assessed by the Mann–Whitney U-tests for continuous variables with non-normal distribution, while the Student’s t-test for continuous variables with normal distribution and the Chi-square test or Fisher’s exact test for categorical variables. The association between each potential risk factor and the presence of reduced D_L_CO in young COPD was determined using logistic regression. The relationship between risk factors and reduced D_L_CO in young COPD was presented as odd ratio (OR). A two-sided *P* < 0.05 was considered statistically significant.

## Result

### Difference in clinical characteristics between young COPD and old COPD

**Fig. 1:**
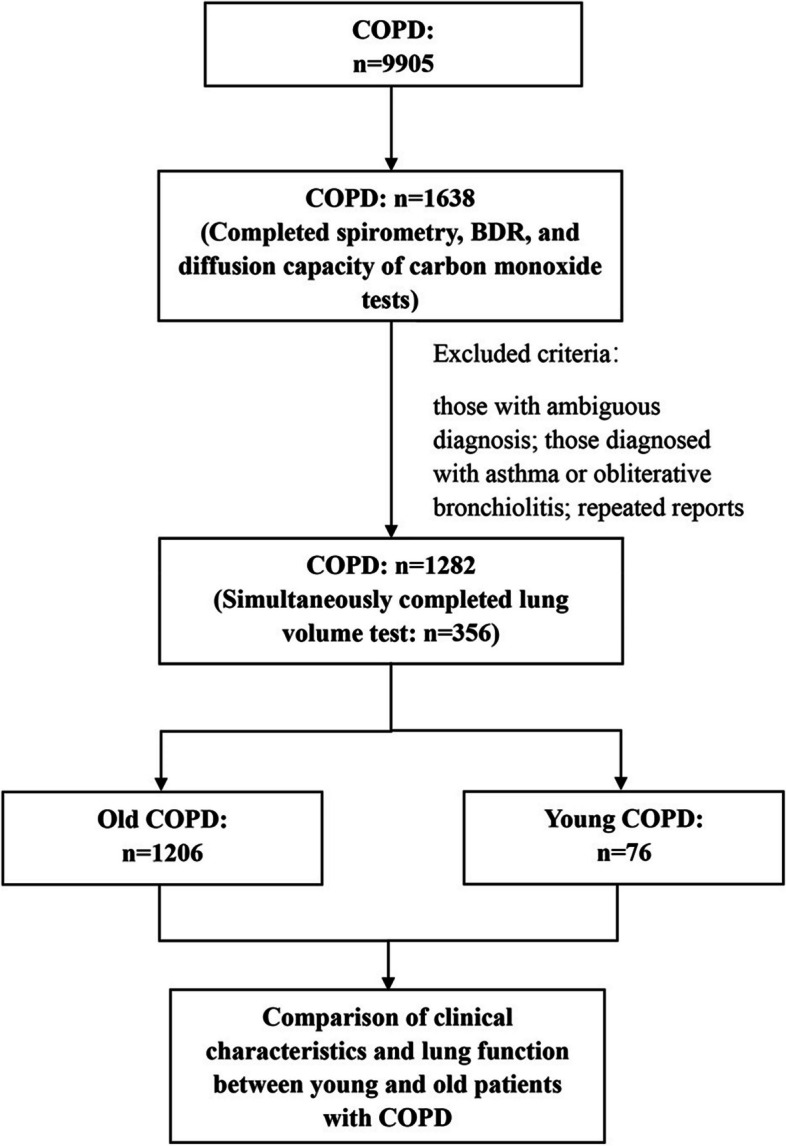
Screening the lung function data of COPD from the database. COPD, chronic obstructive pulmonary disease. BDR, bronchodilator responsiveness

Considering the analysis results after balancing the data were similar to the results based on the unbalanced data, (Supplementary Table 2) which might be more reliable due to their larger sample size, we mainly discussed the analysis results based on the unbalanced data. A total of 1282 patients with COPD were ultimately enrolled in the study, including a young COPD group (*n* = 76) and an old COPD group (*n* = 1206) (Fig. 1). The clinical characteristics were shown in Table 1. The median ages of the young COPD group and old COPD group were 47 years and 66 years, respectively. The proportion of males differed between two groups (73.7% vs. 90.3%, *P* < 0.001). Compared with the old COPD group, the young COPD group demonstrated a significantly lower prevalence of ever or current smokers (25.0% vs. 70.6%, *P* < 0.001) and self-reported smoking history (pack-year) (4.9 ± 11.7 vs. 32.1 ± 32.2, *P* < 0.001), but the young COPD group exhibited a higher incidence of self-reported exposure to air pollution (71.4% vs. 48.8%, *P* = 0.007). Significant intergroup disparities were observed in the prevalence of respiratory symptoms. The young COPD group exhibited a notably lower incidence of individuals experiencing at least one respiratory symptom (44.7% vs. 62.0%, *P* = 0.003), such as cough and dyspnea. Moreover, young COPD had a significantly higher population of mMRC < 2 (89.5% vs. 72.8%, *P* ≤ 0.001).

**Table 1:** Comparison of clinical characteristics between young COPD and old COPD

Variables	Young COPD (*n*=76)	Old COPD (*n*=1206)	*P *value
Age (years)	47.0 (43.3-49.0)	66.0 (61.0-71.0)	＜0.001
Sex			＜0.001
Female	20 (26.3)	117 (9.7)	
Male	56 (73.7)	1089 (90.3)	
BMI (kg/m^2^)	22.4 (18.3-24.6)	21.7 (19.5-24.2)	0.962
BMI (%)			＜0.001
Low BMI (<18.5)	7 (3.6)	213 (19.8)	
Normal BMI (18.5-25)	122 (63.2)	702 (65.2)	
High (≥25)	64 (33.2)	162 (15.0)	
Smoking status			＜0.001
Never smoker	57 (75.0)	354 (29.4)	
Current/Ever smoker	19 (25.0)	852 (70.6)	
Pack-years	4.9±11.7	32.1±32.2	＜0.001
Air pollution exposure	25 (71.4)	391 (48.8)	0.007
History of respiratory diseases	3 (8.1)	38 (4.7)	0.591
Any symptom	34 (44.7)	748 (62.0)	0.003
Cough (%)	23 (30.3)	506 (42.0)	0.045
Sputum (%)	29 (38.2)	569 (47.2)	0.126
Dyspnea (%)	18 (23.7)	490 (40.6)	0.003
mMRC			0.001
< 2	68 (89.5)	878 (72.8)	
≥ 2	8 (10.5)	328 (27.2)	

*Abbreviation: COPD* chronic obstructive pulmonary disease, *BMI* body mass index, *mMRC* modified Medical Research Council. History of respiratory diseases include tuberculosis, bronchiectasis, and interstitial lung disease

### Differences in lung function indices between young COPD and old COPD

**Fig. 2:**
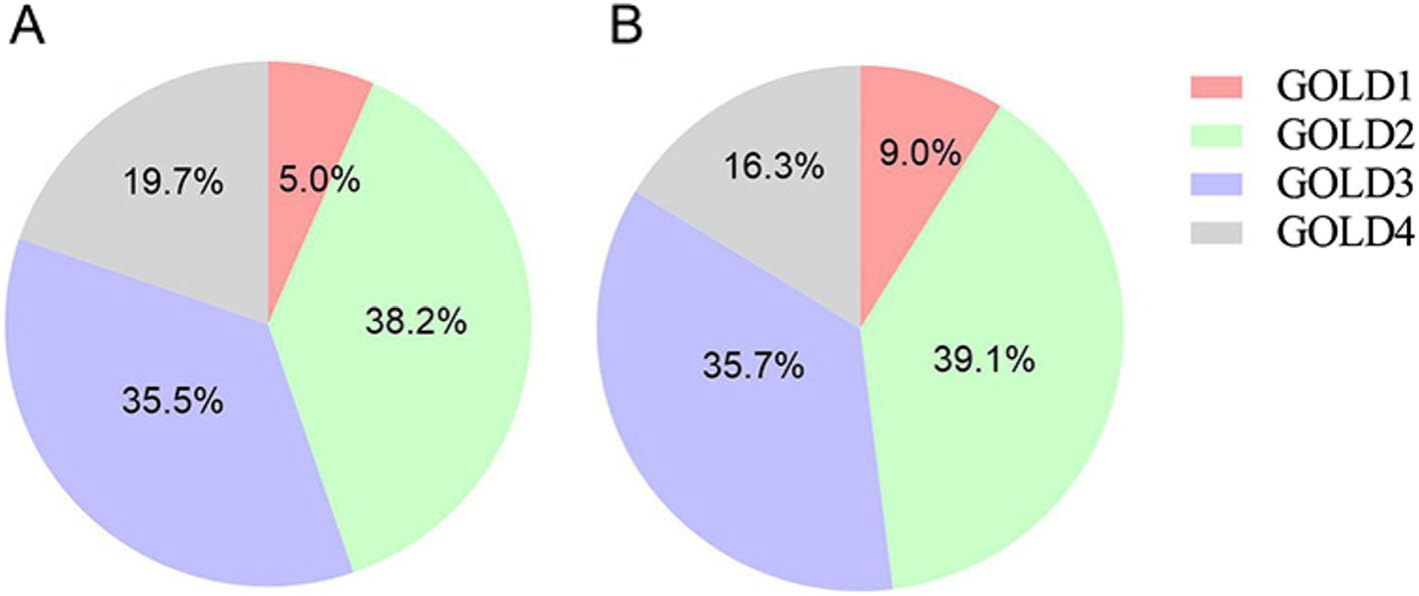
The severity distribution by GOLD categories in young COPD (**A**) and old COPD (**B**). COPD, chronic obstructive pulmonary disease. GOLD, Global Initiative for Chronic Obstructive Lung Disease

**Fig. 3:**
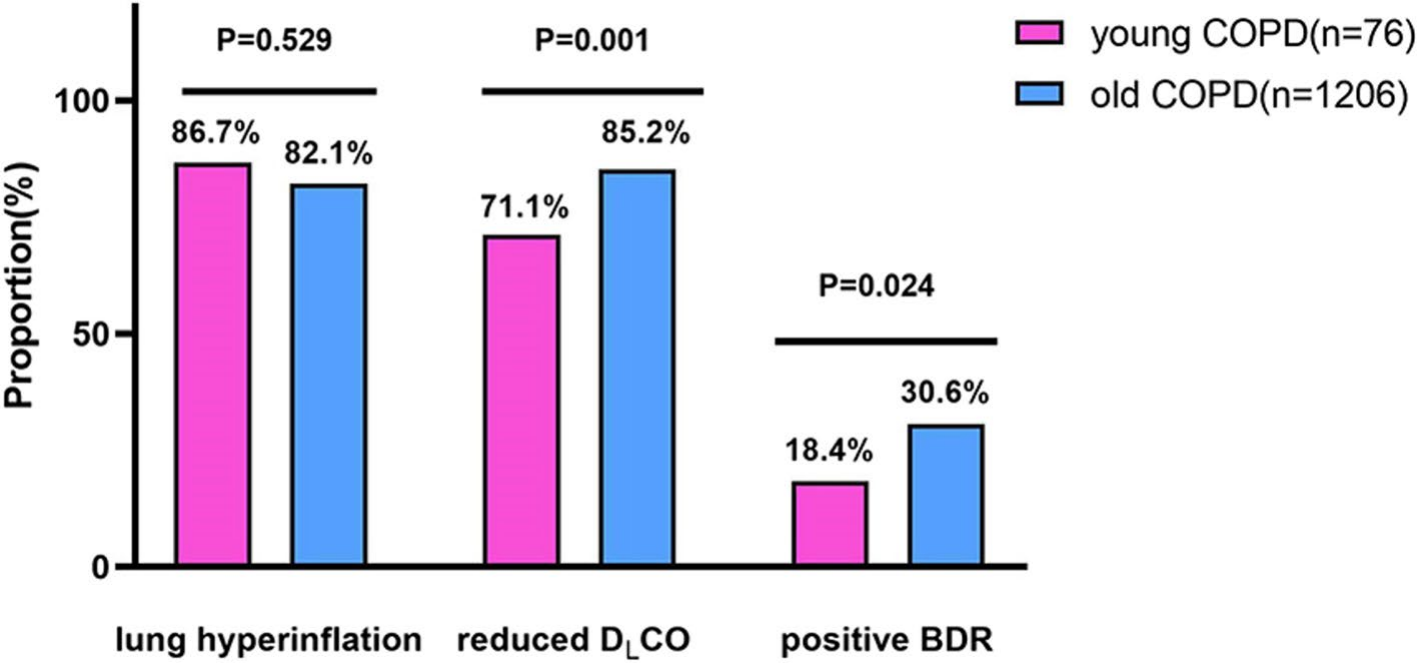
The proportion of lung hyperinflation, reduced D_L_CO and positive BDR between young COPD and old COPD. COPD, chronic obstructive pulmonary disease; BDR, bronchodilator responsiveness; D_L_CO, diffusion capacity of carbon monoxide

**Fig. 4:**
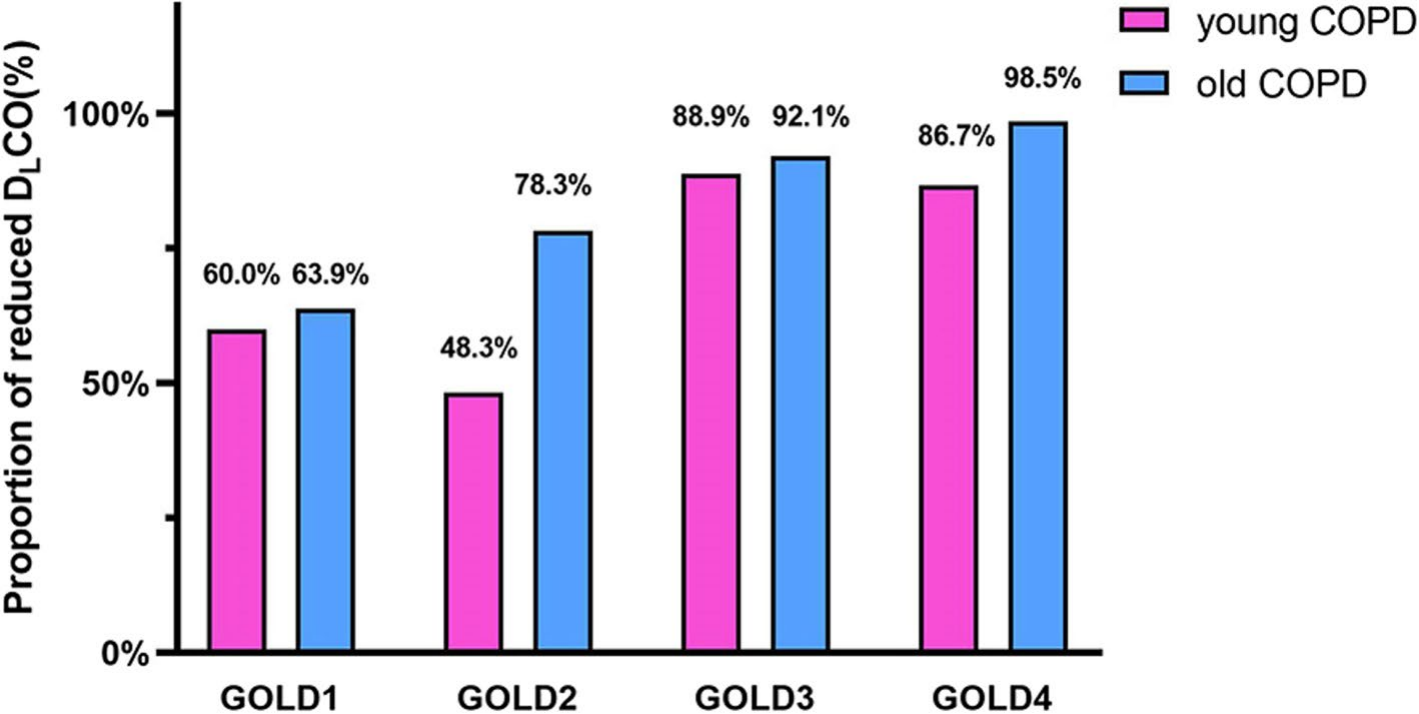
The proportions of reduced D_L_CO among GOLD in young COPD and old COPD. COPD, chronic obstructive pulmonary disease; GOLD, Global Initiative for Chronic Obstructive Lung Disease; D_L_CO, diffusion capacity of carbon monoxide

As shown in Table 2, compared with the old COPD group, the young COPD group had higher median post-BD FEV_1_ (1.4 vs. 1.2 L, *P* = 0.019), post-BD FVC (2.8 vs. 2.7 L, *P* = 0.040), post-BD MMEF (0.7 vs. 0.5 L/s, *P* = 0.001), and D_L_CO (7.2 vs. 4.6, *P*<0.001), while lower median RV (3.0 vs. 3.6 L, *P* = 0.001) and RV/TLC (0.5 vs. 0.6, *P*<0.001). However, the severity distribution by GOLD categories was similar between the two groups (Fig. 2). In addition, the young COPD group showed a markedly lower prevalence of positive BDR compared to the older group(18.4% vs. 30.6%, *P* < 0.001). Unexpectedly, the incidence of lung hyperinflation and reduced D_L_CO in young COPD were up to 86.7% and 71.1%, respectively (Fig. 3). Moreover, the proportions of reduced D_L_CO for GOLD 1–4 in young COPD were 60.0%, 48.3%, 88.9%, and 86.7%, respectively (Fig. 4).

**Table 2:** Comparison of lung function indices between young COPD and old COPD

Variables	Young COPD (*n* = 76)	Old COPD (*n* = 1206)	*P* value
Spirometry (%)			
FEV_1_ (L)			
pre-BD	1.2 (0.9–1.8)	1.1 (0.8–1.6)	0.013
post-BD	1.4 (1.0-1.8)	1.2 (0.9–1.7)	0.019
FEV_1_%pred			
pre-BD	41.6 (29.4–54.8)	44.1 (31.3–60.0)	0.354
post-BD	45.7 (32.1–61.1)	48.5 (35.9–65.3)	0.201
FVC (L)			
pre-BD	2.7 (2.2–3.7)	2.5 (2.0-3.1)	0.007
post-BD	2.8 (2.2–3.9)	2.7 (2.2–3.2)	0.040
FVC%pred			
pre-BD	74.3 (61.8–90.9)	76.2 (64.0-90.4)	0.556
post-BD	77.5 (66.3–91.3)	82.6 (69.9–95.1)	0.101
FEV_1_/FVC			
pre-BD	0.5 (0.4–0.6)	0.5 (0.4–0.6)	0.206
post-BD	0.5 (0.5–0.6)	0.5 (0.4–0.6)	0.121
FEV_1_/FVC%pred			
pre-BD	62.7 (45.5–72.2)	57.1 (45.2–70.6)	0.269
post-BD	65.3 (48.9–77.7)	60.0 (47.1–74.6)	0.097
MMEF (L/s)			
pre-BD	0.5 (0.4–0.8)	0.4 (0.3–0.6)	<0.001
post-BD	0.7 (0.4–0.9)	0.5 (0.3–0.8)	0.001
MMEF%pred			
pre-BD	14.1 (9.1–21.1)	12.8 (8.6–20.3)	0.299
post-BD	17.1 (10.1–24.9)	15.9 (10.4–25.2)	0.754
Lung volume (%)^a^			
RV (L)	3.0 (2.2–3.8)	3.6 (2.9–4.6)	0.001
RV%pred	164.0 (129.5–194.0)	155.3 (126.5-203.1)	0.921
TLC (L)	5.8 (4.6-7.0)	6.4 (5.4–7.2)	0.063
TLC%pred	103.0 (94.0-114.5)	109.3 (94.5-120.6)	0.194
RV/TLC	0.5 (0.5–0.6)	0.6 (0.5–0.7)	<0.001
RV/TLC%pred	157.8 (128.7-178.5)	150.1 (126.0-174.0)	0.442
Diffusing capacity (%)^b^			
D_L_CO	7.2 (5.8–9.2)	4.6 (3.2–6.4)	<0.001
D_L_CO%pred	71.0 (48.1–80.8)	55.1 (40.9–70.5)	<0.001
Reduced DLCO	54 (71.1)	1028 (85.2)	0.001
Mild (60-80%)	28 (51.9)	336 (32.7)	
Moderate (40 -60%)	14 (25.9)	419 (40.8)	
Severe (< 40%)	12 (22.2)	273 (26.6)	

Continuous variables were presented as the median and interquartile range (IQR) and categorical variables were presented as frequency (%)

*Abbreviations: FEV*_1_ Forced expiratory volume in the first second, *FVC* Forced vital capacity, *FEV*_1_/FVC Forced expiratory volume in the first second/forced vital capacity, *MMEF* Maximal-mid expiratory flow, *post-BD* post-bronchodilator responsiveness, *D*_L_CO Diffusion capacity of carbon monoxide, *RV* Residual volume, *TLC* Total lung capacity, *RV/TLC* Ratio of residual volume to total lung capacity

^a^Young COPD (*n* = 31) and Old COPD (*n* = 325)

^b^Young COPD (*n* = 76) and Old COPD (*n* = 1199)

### Risk factors for young COPD with reduced D_L_CO

The clinical characteristics and pulmonary function in young COPD patients with reduced D_L_CO were shown in Supplementary Table 3. Compared with the young COPD with reduced D_L_CO, those with normal D_L_CO had higher BMI and proportion of normal BMI, and exhibited better FEV_1_, FVC, MMEF, RV, and RV/TLC. However, no significant differences were observed in the prevalence of chronic respiratory symptoms between two subgroups. BMI, GOLD, FVC%pred < 80%, and post-BD FEV_1_/FVC were individually included in univariable analysis. Table 3 showed that on univariable logistic regression, the BMI < 18.5 (OR = 10.50, *P* = 0.029) and FVC%pred < 80% (OR = 2.98, *P* = 0.038) were risk factors in young COPD with reduced D_L_CO, but the multivariate logistic analysis results showed that all BMI < 18.5, BMI ≥ 25, GOLD, FVC%pred < 80%, and post-BD FEV_1_/FVC were not associated with reduced D_L_CO in young COPD (*P* > 0.05).

**Table 3:** Univariate and multivariable logistic regression analysis of risk factors for young COPD with diffusing impairment

Variables	Univariable model		Multivariable model	
	Crude OR (95% CI)	*P* value	Adjusted OR (95% CI)	*P* value
Low BMI (ref: Normal BMI)	10.50(1.27–86.93)	0.029	5.57(0.54–57.72)	0.150
High BMI (ref: Normal BMI)	0.43(0.12–1.54)	0.194	0.47(0.12–1.86)	0.279
GOLD2(ref: GOLD1)	0.62(0.09–4.29)	0.630	0.52(0.06–4.57)	0.559
GOLD3(ref: GOLD1)	5.33(0.62–45.99)	0.128	2.53(0.08–78.66)	0.597
GOLD4(ref: GOLD1)	4.33(0.42–44.43)	0.217	1.26(0.01-115.65)	0.919
FVC%pred < 80%	2.98(1.06–8.33)	0.038	1.61(0.40–6.59)	0.506
post-BD FEV_1_/FVC	0.96(0.92-1.00)	0.068	1.01(0.93–1.11)	0.809

*Abbreviation: OR* Odd ratio, *CI* Confidence interval, *BMI* Body mass index, *GOLD* Global initiative for chronic obstructive lung disease,* post-BD* post-Bronchodilator responsiveness

## Discussion

Our study has investigated the clinical characteristics and impairment of lung function in young individuals with COPD. The main findings indicated that young COPD patients showed a similar severity distribution by GOLD categories compared with old COPD patients and that the majority of these young patients had developed lung hyperinflation and reduced DLCO (82.1% and 71.1%, respectively).

We found that the proportion of current or ever smokers in young COPD was 25.0%, significantly lower than that of old COPD (60.6%). However, young COPD showed higher proportions of air pollution exposure and a history of respiratory diseases (including tuberculosis, bronchiectasis, and interstitial lung disease). Therefore, we speculated that in addition to smoking other risk factors may contribute to young COPD, which was supported by a previous study showing genetics, pregnancy and childbirth history, history of respiratory diseases, and air pollution exposure were the risk factors for COPD [22]. Respiratory symptoms are instrumental in motivating individuals to seek medical consultation and serve as significant predictors for early identification of individuals who are at risk for developing COPD. However, we found that most young COPD had fewer chronic respiratory symptoms, which was similar to previous observations [3, 5, 23]. For example, Çolak et al. [23] reported that as much as one-third of individuals with early COPD (FEV_1_/FVC < LLN in smokers under 50 years of age with ≥ 10 pack-years) were asymptomatic. This suggested that a subset of young COPD patients with functional abnormalities might fail to go hospital and undervalue the severity of their condition due to the absence or minimal presence of symptoms. This underscores the importance of lung function testing in the early diagnosis and comprehensive assessment of young COPD patients, particularly for those who do not exhibit clinical signs.

In accordance with previous studies [7, 24], our study showed that the proportion of young COPD distributed among the GOLD categories was similar to old COPD. This might be attributed to two aspects. First, young COPD patients have a low peak lung function in early adulthood due to the following factors, including maternal tobacco smoking, maternal undernutrition, intrauterine growth restriction, preterm birth (< 37 weeks of gestation), bronchopulmonary dysplasia, air pollution exposure, lower respiratory tract infections, and active smoking during adolescence [22, 25, 26]. Second, the accelerated lung function decline in young patients with COPD. Some studies demonstrated that compared with old patients, young COPD had a higher FEV_1_ and more significant space for decline [27]. FEV_1_ declining at a faster rate led to the accelerated progression of COPD. Additionally, we found that more than half of young COPD was GOLD 3–4(56.8%), which was supported by the result of Divo [7]. However, previous studies showed that young COPD was dominated by GOLD 1–2 (96.7% and 98.0%) [3, 5], and few subjects had more than severe airflow limitation. There is a possible explanation that our data was from the medical institution, whose participants were mainly patients with symptoms, while the data resources of other studies were from the National Health and Nutrition Examination Survey, including healthy individuals and mild patients. This suggested that the severity of airflow limitation in young COPD was similar to old COPD and that severe airflow limitation would also occur in young COPD, thus we should attach great attention to lung function screening in young individuals.

It is well known that expiratory flow limitation in patients with COPD can cause an increase in lung volume (hyperinflation) and the decline in lung function varies between patients [28]. To our knowledge, this was the first study to show the differences in lung volume between young COPD and old COPD. In this study, young COPD had lower RV and RV/TLC than the elderly, as possibly the absolute values of RV and RV/TLC increase with aging. However, the medians of RV%pred and RV/TLC%pred in young COPD were higher than those of elderly patients and higher than the normal range, indicating that the gas trapped in young COPD was more severe. It might be explained by the faster decline of FEV_1_ in young COPD patients, leading to aggravative gas trapping [27, 29]. Moreover, our study has revealed an interesting finding that the proportion of lung hyperinflation (RV/TLC%pred > 120%) was highly up to 86.7% in young patients. A previous study showed that lung hyperinflation was observed in mild COPD and the progressive increase in TLC and RV appeared with the worsening airflow limitation during the course of COPD [30]. Therefore, lung hyperinflation may occur from mild to more severe COPD. Moreover, RV/TLC can be used to predict the long-term change of lung function in patients with COPD and is also an independent risk factor for all-cause mortality in COPD. Lung hyperinflation is an independent predictor for frequent exacerbation and links to the quality of life of COPD [31–33]. Therefore, lung volume measurement is warranted in young COPD.

To our knowledge, the structure abnormalities in the airways, alveoli, and pulmonary circulation can lead to the imbalance of ventilation-perfusion distributions, which are considered as main factors to reduced D_L_CO in COPD. In our study, although the severity distribution of GOLD categories and the proportion of lung hyperinflation were similar between young and old COPD, the absolute and percentage predicted values of D_L_CO were significantly better in young COPD than the old patients. We hypothesized that it might be attributed to a discrepancy in the small airway involvement between young and old COPD patients, which was supported by the MMEF being significantly better in the young patients. A previous study has established a correlation between functional small airways disease (fSAD) and low D_L_CO in COPD patients, as fSAD corresponding to pathologic abnormality (including decreased circularity, decreased luminal area, and complete obstruction of terminal bronchioles) impairs gas exchange and leads to reduced D_L_CO [34]. Moreover, a significant correlation between small airway involvement and age, smoking, and pack-years [35, 36]. Therefore, young COPD patients, due to their younger age, lower proportion of smoking, and lower pack-years, have relatively mild damage to small airways, which may lead to less damage to lung diffusing capacity. Additionally, we found that young COPD patients exhibited a more significant improvement of MMEF between the optimal value of baseline and post-BD compared to the old COPD, which was supported by a previous study [36]. This is possible because young COPD patients suffer from less pronounced bronchiolar distortion and inflammation, which results in inhaled drugs being more abundantly deposited in the small airways [37]. Therefore, we speculated that young COPD patients might benefit more from inhaled drug treatment, which needs to be validated with further studies.

Although young COPD had better D_L_CO than the old patients, there was a high proportion of reduced D_L_CO (71.1%) in young COPD. A previous study was similar to our study showing that approximately half of early COPD had abnormal D_L_CO [6]. Of note, reduced D_L_CO also appeared in young COPD with mild airflow restriction (GOLD1-2). Potential explanations for this might be that reduced D_L_CO was associated with other risk factors rather than smoking in early life [38, 39]. For example, the D_L_CO was persistently reduced in extremely preterm (EP) subjects, which sustained from mid-childhood to adulthood, with no signs of pubertal catch-up growth at 25 years, while the disruption of alveolar growth associated with EP birth may be linked to early-onset COPD in adult life [39]. Therefore, the association between the reduced D_L_CO and the young COPD remains unclear, which needs to be explored with further studies.

In the present study, we observed that young COPD with reduced D_L_CO had lower BMI and worse lung function parameters (including FEV1, FVC, RV, and RV/TLC). The univariable logistic regression showed that the low BMI and FVC%pred < 80% were associated with reduced D_L_CO. This was supported by previous studies [40, 41]. Lim et al. revealed that low BMI is linearly correlated with reduced D_L_CO [40]. In addition, the decreased FVC in the patients with COPD was caused by hyperinflation or air trapping, [42] which also worsened the effectiveness of gas exchange and declined D_L_CO. However, the multivariate logistic analysis showed that all the above influence factors had no statistical significance. This biased result might be due to the small sample, thus further exploration is needed to expand the sample size in future studies.

We believe the most important aspect of this work was the first time to collect the indices of multiple lung function tests, including spirometry, bronchodilator responsiveness testing, lung volume measurements, and diffusion capacity of carbon monoxide. However, we acknowledge several limitations of this study. First, the study did not enroll healthy young individuals (20–50 years) as the control group and did not explore differences in lung function between young patients without and with COPD. Second, it was a cross-sectional study that failed to observe the changes in long-term lung function and prognosis in young COPD patients. However, the long-term follow-up, including the impairment of lung function and the characteristics of disease progression, was critical for researching the treatment in young COPD, and we hope to improve this limitation in future research. Finally, we did not obtain other important clinical characteristics, such as detailed medical history, laboratory indicators, imaging features, and so on. Due to the fact that the questionnaire involved in this study was a routine questionnaire before lung function test and not specifically designed for COPD, the information provided was very limited. Additionally, some outpatients did not undergo laboratory examinations and chest imaging scans in our hospital. In future research, we will collect data from multiple centers for screening, hoping to better address this issue.

## Conclusion

In conclusion, despite having fewer respiratory symptoms and better FEV_1_, FVC, RV/TLC, and D_L_CO compared with old COPD, young COPD have a similar disease severity distribution by GOLD categories. In addition, most of these young people have shown lung hyperinflation and reduced D_L_CO. Our findings support the need to screen airway obstruction in young COPD individuals and the preventive and treatment strategies were greatly important for improving progression and outcomes.

### Supplementary Information

Supplementary Material 1.

## Data Availability

The data presented in this study are available on request from the corresponding author.

## Abbreviations

COPD: Chronic obstructive pulmonary disease
BDR: Bronchodilator responsiveness
FEV_1_: Forced expiratory volume in 1 s
D_L_CO: Diffusing capacity of the lung for carbon monoxide
RV: Residual volume
TLC: Total lung capacity
RV/TLC: Residual volume to total lung capacity ratio
GOLD: Global initiative for chronic obstructive lung disease
FVC: Forced vital capacity
FEV_1_/FVC: Forced expiratory volume in 1 second to forced vital capacity ratio
PFT: Pulmonary function testing
mMRC: Modified Medical Research Council
IQR: Interquartile range
ERS/ATS: The American Thoracic Society and the European Respiratory Society
MMEF: Maximal- mid expiratory flow
SD: Standard deviation
BMI: Body mass index
OR: Odd ratio
CI: Confidence interval
LLN: Lower limit of normal
fSAD: Functional small airways disease
EP: Extremely preterm
