# Supplementary material for: Comprehensive appraisal of lung function in young COPD patients: a single center observational study
Source: BMC Pulm Med. 2024 Jul 24;24:358. doi: 10.1186/s12890-024-03165-9 (PMC11267774; doi:10.1186/s12890-024-03165-9)
Supplement: Supplementary file 1 — Supplementary Material 1. [file 12890_2024_3165_MOESM1_ESM.docx]

**Supplementary Table 1** The details of the questionnaire for lung function testing.

| **Questionnaire For Lung Function Testing** | |
| --- | --- |
| **Demographic characteristics** |  |
| - ID: | - Name: |
| - Gender: | - Date of Birth: |
| - Height (CM): | - Weight (KG): |
| - Contact Phone Number: | - Applying Department: |
| - Occupation: | - Medical Record Number: |
| **Vital signs** |  |
| - Blood Pressure (mmHg): | - Heart Rate(beats per minute): |
| - Body Temperature (°C): | - Oxygen Saturation (SpO2): |
| - Respiratory Rate (breaths per minute): | |
| **General condition** |  |
| - Smoking History: □ Yes □ No   If yes, for how long?  How many packs of cigarettes do you smoke per day?  If you have quit smoking, when did you quit? | |
| - Cough: □ Yes □ No   If yes, for how long? [Duration of cough]  Do you often cough in the late night or early morning: □ Yes □ No | |
| - Wheezing: □ Yes □ No   If yes, for how long? [Duration of wheezing] | |
| - Sputum: □ Yes □ No   If yes, describe the color and consistency of your sputum. | |
| - Dyspnea (shortness of breath): □ Yes □ No   If yes, describe the severity and duration.  Severity Level: (Please follow the standard below)  1. Able to walk on flat ground at a normal pace compared to peers, but cannot do so when climbing stairs or slopes.  2. Unable to walk on flat ground at a normal pace compared to peers, but can walk long distances by oneself.  3. Unable to walk more than 100 meters on flat ground.  4. Short of breath with slight activity or at rest. | |
| **Medical History** |  |
| - History of Respiratory Diseases (including history of thoracic surgery):   □ Yes □ No  If yes, describe the specific condition(s). | |
| - History of Tuberculosis: □ Yes □ No | |
| - Cardiovascular History: □ Yes □ No   If yes, describe the specific condition(s). | |
| - Have you ever been affected by exposure to polluted air, dust, or changes in gas: □ Yes □ No | |
| **Clinical Diagnosis** |  |
| - New: | - Other: |
| **Surgical Information** |  |
| - Intended Surgical Procedure including site and technique | |
| **Recent Medication Use** |  |
| - Bronchodilators   □ Yes □ No  Medication Name:  Dosage:  Last Time Used: | - Allergy Medications:   □ Yes □ No  Medication Name:  Dosage:  Last Time Used: |
| - Other Medications:   □ Yes □ No  Medication Name:  Dosage:  Last Time Used: |  |
| **Recent Imaging Information** |  |
| - Date: | - Results: |

**Supplementary Table 2** Comparison of characteristics and lung function between young and old patients with COPD (after balancing the data).

| **Variables** | **Young COPD(n=76)** | **Old COPD(n=152)** | ***P* value** |
| --- | --- | --- | --- |
| Age (years) | 47 (43.3-49.0) | 66.0 (61.0-72.0) | <0.001 |
| Sex (%) |  |  | 0.009 |
| Female | 22 (27.3) | 19 (12.5) |  |
| Male | 56 (73.7) | 133 (87.5) |  |
| BMI (kg/m^2^) | 22.4 (18.3-24.6) | 21.4 (19.2-24.1) | 0.664 |
| BMI (%) |  |  | 0.468 |
| <18.5 | 15 (19.7) | 24 (15.8) |  |
| 18.5-25 | 37 (48.7) | 87 (57.2) |  |
| ≥25 | 24 (31.6) | 41 (27.0) |  |
| Smoking status (%) |  |  | <0.001 |
| Never smoker | 57 (75.0) | 40 (26.3) |  |
| Current/Ever smoker | 19 (25.0) | 112 (73.7) |  |
| Pack-years | 4.9±11.7 | 29.6±29.5 | <0.001 |
| Air pollution exposure (%) | 25 (71.4) | 47 (46.5) | 0.011 |
| History of respiratory diseases (%) | 3 (8.1) | 4 (4.1) | 0.613 |
| Any symptoms (%) | 34 (44.7) | 91 (59.9) | 0.030 |
| Cough (%) | 23 (30.3) | 61 (40.1) | 0.145 |
| Sputum (%) | 29 (38.2) | 66 (43.4) | 0.447 |
| Dyspnea (%) | 18 (23.7) | 57 (37.5) | 0.036 |
| mMRC (%) |  |  | 0.002 |
| <2 | 68 (89.5) | 109 (71.7) |  |
| ≥2 | 8 (10.5) | 43 (28.3) |  |
| Spirometry (%) |  |  |  |
| FEV_1_(L) |  |  |  |
| pre-BD | 1.2 (0.9-1.8) | 1.1 (0.8-1.5) | 0.019 |
| post-BD | 1.4 (1.0-1.8) | 1.2 (0.9-1.7) | 0.041 |
| FEV_1_%pred |  |  |  |
| pre-BD | 41.6 (29.4-54.8) | 44.4 (32.7-58.8) | 0.387 |
| post-BD | 45.7 (32.1-61.1) | 48.6 (37.2-65.2) | 0.176 |
| FVC (L) |  |  |  |
| pre-BD | 2.7 (2.2-3.7) | 2.5 (2.0-3.0) | 0.015 |
| post-BD | 2.8 (2.2-3.9) | 2.7 (2.2-3.3) | 0.073 |
| FVC%pred |  |  |  |
| pre-BD | 74.3 (61.8-90.9) | 76.3 (64.0-91.7) | 0.502 |
| post-BD | 77.5 (66.3-91.3) | 85.0 (69.5-95.6) | 0.061 |
| FEV_1_/FVC |  |  |  |
| pre-BD | 0.5 (0.4-0.6) | 0.5 (0.4-0.6) | 0.274 |
| post-BD | 0.5 (0.4-0.6) | 0.5 (0.4-0.6) | 0.219 |
| FEV_1_/FVC%pred |  |  |  |
| pre-BD | 61.6 (43.9-72.1) | 53.1 (40.1-68.2) | 0.095 |
| post-BD | 64.0 (46.6-77.5) | 55.6 (40.1-71.8) | 0.036 |
| MMEF (L/s) |  |  |  |
| pre-BD | 0.5 (0.4-0.8) | 0.4 (0.3-0.6) | <0.001 |
| post-BD | 0.7 (0.4-0.9) | 0.5 (0.3-0.8) | 0.008 |
| MMEF%pred |  |  |  |
| pre-BD | 14.1 (9.1-21.1) | 12.2 (8.7-19.4) | 0.307 |
| post-BD | 17.1 (10.1-24.9) | 15.7 (10.9-26.1) | 0.993 |
| Lung volume (%)^a^ | |  |  |
| RV (L) | 3.0 (2.2-3.8) | 3.7 (2.9-4.9) | 0.003 |
| RV%pred | 164.0 (129.5-194.0) | 170.3 (133.4-204.8) | 0.537 |
| TLC (L) | 5.8 (4.6-7.0) | 6.3 (5.3-7.2) | 0.157 |
| TLC%pred | 103.0 (94.0-114.5) | 112.8 (101.3-120.0) | 0.049 |
| RV/TLC | 0.5 (0.5-0.6) | 0.6 (0.6-0.7) | <0.001 |
| RV/TLC%pred | 157.8 (128.7-178.5) | 158.6 (133.2-172.1) | 0.905 |
| Diffusing capacity (%)^b^ |  |  |  |
| D_L_CO | 7.2 (5.8-9.2) | 4.8 (3.4-6.2) | <0.001 |
| D_L_CO%pred | 71.0 (48.1-80.8) | 58.4 (43.2-71.3) | 0.003 |
| Reduced D_L_CO (%) | 54 (71.1) | 132 (86.8) | 0.004 |
| Mild | 28 (51.9) | 54 (40.9) |  |
| Moderate | 14 (25.9) | 47 (35.6) |  |
| Severe | 12 (22.2) | 31 (23.5) |  |

Continuous variables were presented as the median and interquartile range (IQR) and categorical variables were presented as frequency (%). a: Young COPD (n = 31) and Old COPD (n = 50); b: Young COPD (n = 76) and Old COPD (n = 152).

**Abbreviations:** COPD, chronic obstructive pulmonary disease; BMI, body mass index; mMRC, modified Medical Research Council; FEV_1_, forced expiratory volume in the first second; FVC, forced vital capacity; FEV_1_/FVC, forced expiratory volume in the first second/forced vital capacity; MMEF, maximal mid-expiratory flow; post-BD, post-bronchodilator responsiveness; D_L_CO, diffusion capacity of carbon monoxide; RV, residual volume; TLC, Total lung capacity; RV/TLC, ratio of residual volume to total lung capacity.

**Supplementary Table 3** Comparison of characteristics and lung function between normal D_L_CO group and reduced D_L_CO group in young COPD

| **Variables** | **Normal D_L_CO**  **(n=22)** | **Reduced D_L_CO**  **(n=54)** | ***P* value** |
| --- | --- | --- | --- |
|  |  |  |  |
| Age (years) | 47.0 (45.0-49.3) | 46.0 (43.0-49.0) | 0.238 |
| Sex (%) |  |  | 0.650 |
| Female | 5 (22.7) | 15 (27.8) |  |
| Male | 17 (77.3) | 39 (72.2) |  |
| BMI (kg/m^2^) | 24.5 (23.0-26.6) | 20.5 (17.7-23.3) | <0.001 |
| BMI (%) |  |  | 0.005 |
| <18.5 | 1 (4.8) | 21 (39.6) |  |
| 18.5-25 | 13 (61.9) | 26 (49.1) |  |
| ≥25 | 7 (33.3) | 6 (11.3) |  |
| Smoking status (%) |  |  | 0.381 |
| Never smoker | 19 (86.4) | 45 (83.3) |  |
| Current/Ever smoker | 3 (13.6) | 9 (16.7) |  |
| Pack-years | 3.9±10.9 | 3.3±10.0 | 0.841 |
| Air pollution exposure (%) | 10 (90.9) | 15 (62.5) | 0.089 |
| History of respiratory diseases (%) | 1 (8.3) | 2 (8.0) | 0.704 |
| Any symptom (%) | 9 (40.9) | 25 (46.3) | 0.668 |
| Cough (%) | 6 (27.3) | 17 (31.5) | 0.717 |
| Sputum (%) | 7 (31.8) | 22 (40.7) | 0.468 |
| Dyspnea (%) | 7 (31.8) | 11 (20.4) | 0.287 |
| mMRC (%) |  |  | 0.795 |
| <2 | 20 (90.9) | 48 (88.9) |  |
| ≥2 | 2 (9.1) | 6 (11.1) |  |
| Spirometry (%) |  |  |  |
| FEV_1_(L) |  |  |  |
| pre-BD | 1.7 (1.3-2.4) | 1.2 (0.9-1.5) | 0.005 |
| post-BD | 1.8 (1.4-2.5) | 1.3 (0.9-1.7) | 0.004 |
| FEV_1_%pred |  |  |  |
| pre-BD | 53.6 (46.4-65.4) | 38.1 (27.6-50.0) | 0.002 |
| post-BD | 57.4 (47.9-68.3) | 42.8 (29.9-53.4) | 0.002 |
| FVC (L) |  |  |  |
| pre-BD | 3.6 (2.4-4.0) | 2.6 (2.0-3.3) | 0.040 |
| post-BD | 3.5 (2.6-4.2) | 2.7 (2.2-3.7) | 0.034 |
| FVC%pred |  |  |  |
| pre-BD | 87.4 (68.0-98.7) | 70.3 (60.3-83.7) | 0.008 |
| post-BD | 89.5 (75.9-97.6) | 76.1 (60.3-87.3) | 0.008 |
| FEV_1_/FVC |  |  |  |
| pre-BD | 0.6 (0.5-0.6) | 0.5 (0.4-0.6) | 0.030 |
| post-BD | 0.6 (0.5-0.7) | 0.5 (0.4-0.6) | 0.046 |
| FEV_1_/FVC%pred |  |  |  |
| pre-BD | 70.6 (56.8-74.4) | 59.6 (43.8-71.9) | 0.029 |
| post-BD | 75.3 (62.3-80.5) | 61.6 (47.1-76.9) | 0.031 |
| MMEF (L/s) |  |  |  |
| pre-BD | 0.7 (0.5-1.1) | 0.5 (0.3-0.7) | 0.021 |
| post-BD | 1.0 (0.7-1.5) | 0.6 (0.4-0.8) | 0.005 |
| MMEF%pred |  |  |  |
| pre-BD | 18.0 (12.7-26.1) | 12.3 (8.9-19.5) | 0.022 |
| post-BD | 24.9 (18.0-38.0) | 16.1 (9.7-20.4) | 0.003 |
| Lung volume restriction (%) | 14 (63.6) | 20 (37.0) | 0.034 |
| Lung volume (%)^a^ |  |  |  |
| RV(L) | 2.2 (1.9-2.6) | 3.2 (2.6-3.9) | 0.018 |
| RV%pred | 116.1 (88.0-129.5) | 178.1 (147.6-194.6) | 0.006 |
| TLC(L) | 5.6 (4.5-6.4) | 5.8 (4.7-7.0) | 0.705 |
| TLC%pred | 95.0 (87.0-100.8) | 105.5 (97.3-115.6) | 0.065 |
| RV/TLC | 0.4 (0.3-0.5) | 0.5 (0.5-0.6) | 0.002 |
| RV/TLC%pred | 122.1 (105.8-144.7) | 167.1 (144.2-188.1) | 0.004 |

Continuous variables were presented as the median and interquartile range (IQR) and categorical variables were presented as frequency (%). a: Normal D_L_CO (n = 7) and Reduced D_L_CO (n = 14).

**Abbreviations:** COPD, chronic obstructive pulmonary disease; BMI, body mass index; mMRC, modified Medical Research Council; FEV_1_, forced expiratory volume in the first second; FVC, forced vital capacity; FEV_1_/FVC, forced expiratory volume in the first second/forced vital capacity; MMEF, maximal mid-expiratory flow; post-BD, post-bronchodilator responsiveness; D_L_CO, diffusion capacity of carbon monoxide; RV, residual volume; TLC, Total lung capacity; RV/TLC, ratio of residual volume to total lung capacity.
